# Correlation between inflammatory biomarkers and metabolic disorders in HIV infected patients undergoing antiretroviral therapy

**DOI:** 10.1186/1471-2334-13-S1-O33

**Published:** 2013-12-16

**Authors:** Raluca Mihăilescu, Victoria Aramă, Cătălin Tilişcan, Daniela Munteanu, Viorica Leoveanu, Mihaela Rădulescu, Adriana Hristea, Cristina Popescu, Ruxandra Moroti, Violeta Molagic, Raluca Năstase, Loredana Benea, Ana Maria Tudor, Mihai Lazăr, Anca-Ruxandra Negru, Irina Lăpădat, Ligia Ionescu, Mirela Cernat, Georgeta Jugănaru, Doina Cristea, Adriana Manea, Adrian Streinu-Cercel, Daniela Adriana Ion, Sorin Ștefan Aramă

**Affiliations:** 1 National Institute for Infectious Diseases “Prof. Dr. Matei Balş”, Bucharest, Romania; 2 Carol Davila University of Medicine and Pharmacy, Bucharest, Romania

## Background

The objective of the present study was to evaluate the correlation between inflammatory biomarkers and metabolic syndrome and insulin-resistance in HIV seropositive patients, undergoing antiretroviral therapy (cART).

## Methods

We performed a cross-sectional study, conducted as part of the research grant PNCDI2 no.62077/2008 on HIV-infected patients undergoing cART, recruited in a tertiary care hospital, between 2009-2011. BioSource EASIA (Enzyme Amplified Sensitivity Immunoassay) quantified tumor necrosis factor-alpha (TNF alpha), interleukin-6 (IL6), monocyte chemotactic protein 1 (MCP1), high-sensitivity C-reactive protein (hsCRP).

## Results

We included 101 patients, characterized by: M:F ratio 1.3; median age of 31 years. Undetectable HIV viremia was found in 75.2% of the subjects. The detectable viremia group had median of age 20 years versus 31, CD4 334/cmm versus 546/cmm (p=0.007) and 4 therapeutic combinations versus 1 in undetectable patients (p=0.000). Pathological values of MCP 1 were found 2 times more frequently in the detectable group. Prevalence of metabolic syndrome was 12 versus 18.4% while prevalence of insulin-resistance was 50 versus 64.8% in detectable versus undetectable patients, p>0.05. Each logarithmical increase of MCP 1 augments the risk of metabolic syndrome by 3.4 times (CI95% 1.2-10, p=0.03) and the risk of insulin-resistance by 2.9 times (CI95% 1.2-7.7, p=0.03).

## Conclusion

The prevalence of metabolic syndrome and insulin-resistance are moderate to high. They seem higher in the undetectable group than in the detectable one, probably because of the one-decade difference of age and of the preponderance of overweight males in the undetectable group. Metabolic disorders are not related to viral replication, but probably cART impacts on adipocytes in all patients. MCP 1 is correlated with the presence of both metabolic syndrome and insulin-resistance.

